# Competing endogenous network analysis identifies lncRNA Meg3 activates inflammatory damage in UVB induced murine skin lesion by sponging miR-93-5p/epiregulin axis

**DOI:** 10.18632/aging.102483

**Published:** 2019-11-24

**Authors:** Nan Zhang, Zhou Zhong, Yujia Wang, Li Yang, Fengbo Wu, Cheng Peng, Wei Huang, Gu He

**Affiliations:** 1 State Key Laboratory of Biotherapy, Department of Orthopaedic Surgery, West China Hospital, Sichuan University, Chengdu 610041, China; 2 Key Laboratory of Southwestern Chinese Medicine Resources, School of Pharmacy, Chengdu University of Traditional Chinese Medicine, Chengdu 611137, China

**Keywords:** ultraviolet, lncRNA Meg3, ceRNA, inflammatory damage, skin

## Abstract

In this study, we obtained the RNA expression data of murine skin tissues of control, and UVB irradiated groups. After the re-annotation of lncRNAs, a gene expression similarity analysis was done by WGCNA. The target mRNA prediction of lncRNAs, miRNAs, and ceRNA regulatory networks were constructed by five lncRNAs, 14 miRNAs and 54 mRNAs, respectively. Based on the ceRNA network of UVB-induced skin lesions, it was evident that the dysregulation of Meg3 has critical effects on the UVB-induced inflammatory lesion of murine skin tissues. The overexpression of Meg3 after UVB irradiation was observed in primary murine skin fibroblasts, and the up-regulated Meg3 expression was related to the activation of the inflammatory cytokines. These functional experiments demonstrated that the RNA silencing of Meg3 in murine skin fibroblasts could suppress the expression of the cytokines (*in vitro*) and UVB-induced skin lesions (*in vivo)*. Moreover, the Meg3 functioned as a competing endogenous RNA (ceRNA) that acted as a sponge for miR-93-5p and thereby modulated the expression of Epiregulin (Ereg). Our results proved that Meg3 was involved in UVB-induced skin inflammation and that the ceRNA networks, which includes miR-93-5p and Ereg, could prove to be a potential therapeutic target for UVB-induced skin damage.

## INTRODUCTION

Skin exposure to ultraviolet radiation (UV) is necessary to enhance vitamin D production; however, excessive exposure to UV cause skin malignancies [1–3]. The correlation between excessive UV irradiation and UV-induced skin lesions such as inflammation, dysfunction of the epidermal barrier, skin aging, carcinogenesis, and melanoma are not known [4–7]. Typically, the lncRNAs (long non-coding RNA) act as a pivotal regulator in a panel of inflammatory diseases, including diabetes, rheumatoid arthritis, psoriasis, systemic lupus, and erythematosus [18, 19]. However, the involvement of lncRNAs in UV-induced skin inflammation remains unclear. Besides transcriptional noise, the lncRNA with the sequence lengths longer than 200 nucleotides could regulate gene expression *via* a cis- or trans- mechanism, which allows it to associate with RNA and de-stabilizes the target proteins [8–17]. MicroRNA (miRNA) also exerts a regulatory role in skin inflammations by mediating target mRNA degradation or inhibiting the mRNA translation [20–25]. However, the latest research indicates that the ceRNA (competing endogenous RNA) network constitute the key regulatory mechanism in the pathogenesis and development of skin disorders. The lncRNA maternally expressed gene 3 (human transcript named MEG3 and mouse transcript named Meg3) located in the chromosome 14q and chromosome 12 in the human and mouse genome, respectively could identify chromatin and initiate interaction with the RNA-DNA complex, PRC2 (polycomb repressive complex 2), and various target genes [26–29]. Most miRNAs could suppress the MEG3 expression *via* post-transcription regulation which competes with the endogenous RNA mechanism [30–34]. MEG3 could also activate or inhibit multiple signaling pathways, i.e., p53, TGFβ, Rb, and EZH2; and hence, the dysregulation of MEG3 expression was typical to solid tumors, inflammation, and autoimmune disease [34–38].

For the first time in our study, we identified the differentially expressed mRNAs, lncRNAs, and miRNAs in untreated, and UVB irradiated murine dorsal skin tissues (GSE80427 and 80428 with reannotation of lncRNAs). The WGCNA (weighted correlation network analysis) and ceRNA network construction was performed following an enrichment analysis from gene ontology (GO) and Kyoto Encyclopedia of Genes and Genomes (KEGG) pathways to discover potential therapeutic targets for skin inflammation induced by UVB irradiation. Furthermore, we confirmed *in vivo* and *in vitro* up-regulated status of lncRNA Meg3 after a UVB irradiation. Following the interactions with miR-93-5p, the skin inflammatory responses were activated in murine skin fibroblasts by lncRNA Meg3. Correspondingly, the ceRNA mechanism revealed that the UVB induced inflammatory skin lesions were dependent on Meg3/miR-93-5p/Ereg axis.

## RESULTS

### Microarray re-annotation and differential expression RNAs analysis

**Figure 1:**
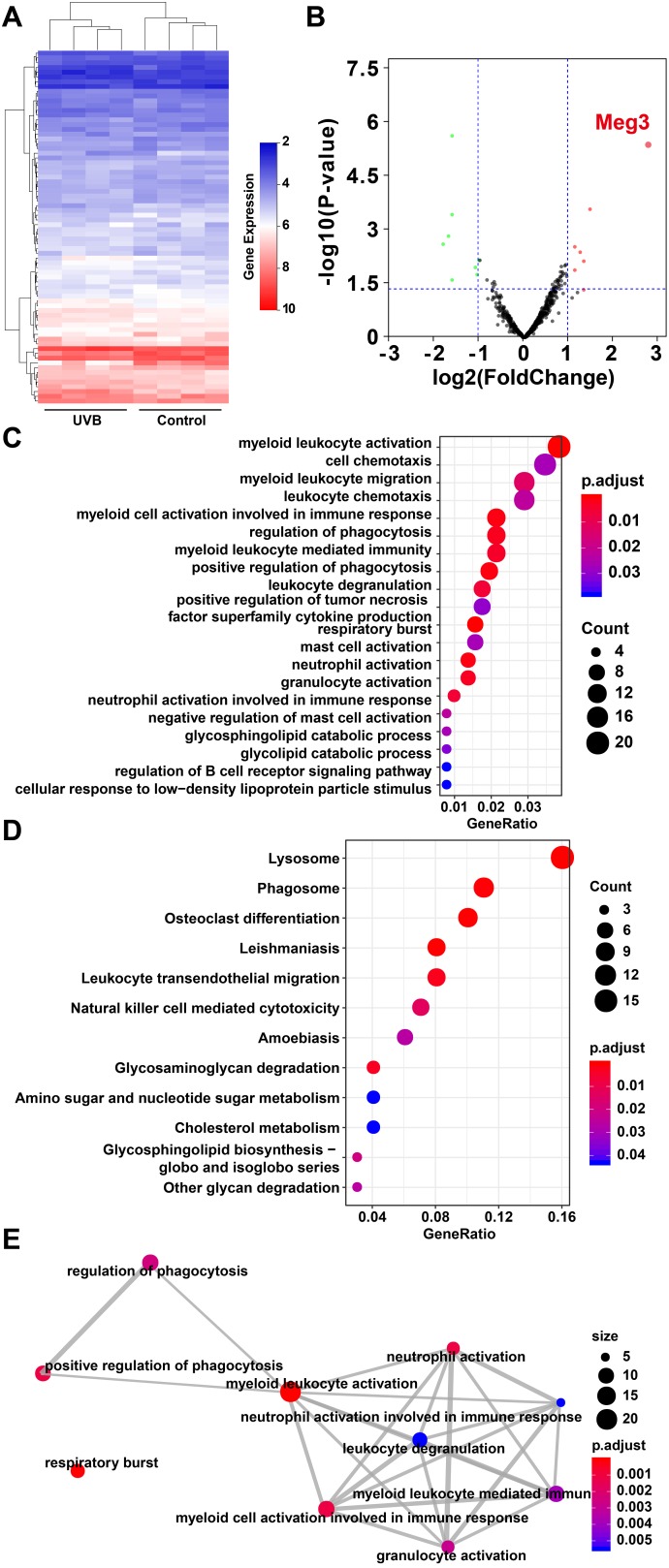
(**A**) Heat map of lncRNAs expression profiles of normal and UVB irradiated murine dorsal skin tissue groups. Red represents up-regulated lncRNAs and blue represents down-regulated lncRNAs. (**B**) Volcano plots of lncRNAs for normal and UVB irradiated murine dorsal skin tissue groups. The horizontal axis represents fold change (log 2) and the vertical axis is P value (−log 10). Red points (fold change > 1) indicate up-regulated lncRNAs, green points (fold change < −1) indicate down-regulated lncRNAs. Gene ontology analysis (**C**) and KEGG enrichment (**D**) of differentially expressed lncRNAs in normal and UVB irradiated murine dorsal skin tissue groups. The horizontal axis represents the proportion of those genes accounted for in all the annotated genes, the left side of the vertical axis represents the annotation terms. Bubble scale represents number of genes in each term; depth of bubble color represents p value. (**E**) The annotation terms are displayed as an interaction network by using the Reactome pathways. Bubble scale represents number of genes; depth of bubble color represents p value.

The expression profiles of RNAs in four control and UVB irradiated murine skin tissues (12-weeks) were retrieved from the GEO database (Gene Expression Omnibus, https://www.ncbi.nlm.nih.gov/geo/ GSE80427 and GSE80428) and analyzed by following the reported literature [39–41]. The gene expression microarray platform of GSE80427 was Affymetrix Mouse Gene 1.0 ST Array. Typically, 655 lncRNAs were identified by the default microarray annotation files. Based on the Gencode_M22 (GRCm38.p6) annotation, the lncRNAs of (GSE80427) were re-annotated, and a total of 1,854 lncRNAs were identified with at least three independent probes. The differential expressed lncRNAs, mRNAs, and miRNAs were determined by the limma method after normalization. More specifically, 7 significantly up-regulated lncRNAs, 8 down-regulated lncRNAs, 24 significantly up-regulated mRNAs, 160 down-regulated mRNAs in GSE80427, 51 significantly up-regulated miRNAs and 54 down-regulated miRNAs in GSE80428 were determined, respectively. Figure 1A and 1B displayed the heatmap of clustered DE-lncRNAs and the distribution of all the annotated lncRNAs by a two-dimension logarithmic scale, i.e., -log10 (p-values) and log2 (fold change, FC) in a volcano map. LncRNA Meg3 was the most up-regulated lncRNA with the highest statistical significance, and the corresponding results on the mRNAs and miRNAs were summarized in Supplementary Figure 1.

The gene modules related to differential expressed lncRNAs and mRNAs were enriched by gene ontology (GO) annotation [42] and Kyoto Encyclopedia of Genes and Genomes (KEGG) [43] respectively. As shown in Figure 1C, the myeloid leukocyte, cellulose chemotaxis, and myeloid leukocyte migration were enriched. [44] The relationship among the top pathways was analyzed by the Reactome database (as shown in Figure 1E) i.e. the myeloid leukocyte, and cellular activation involved in the immune responses were the centers for all the enriched pathways [45].

### Identification of gene modules by weighted gene co-expression network analysis (WGCNA)

**Figure 2:**
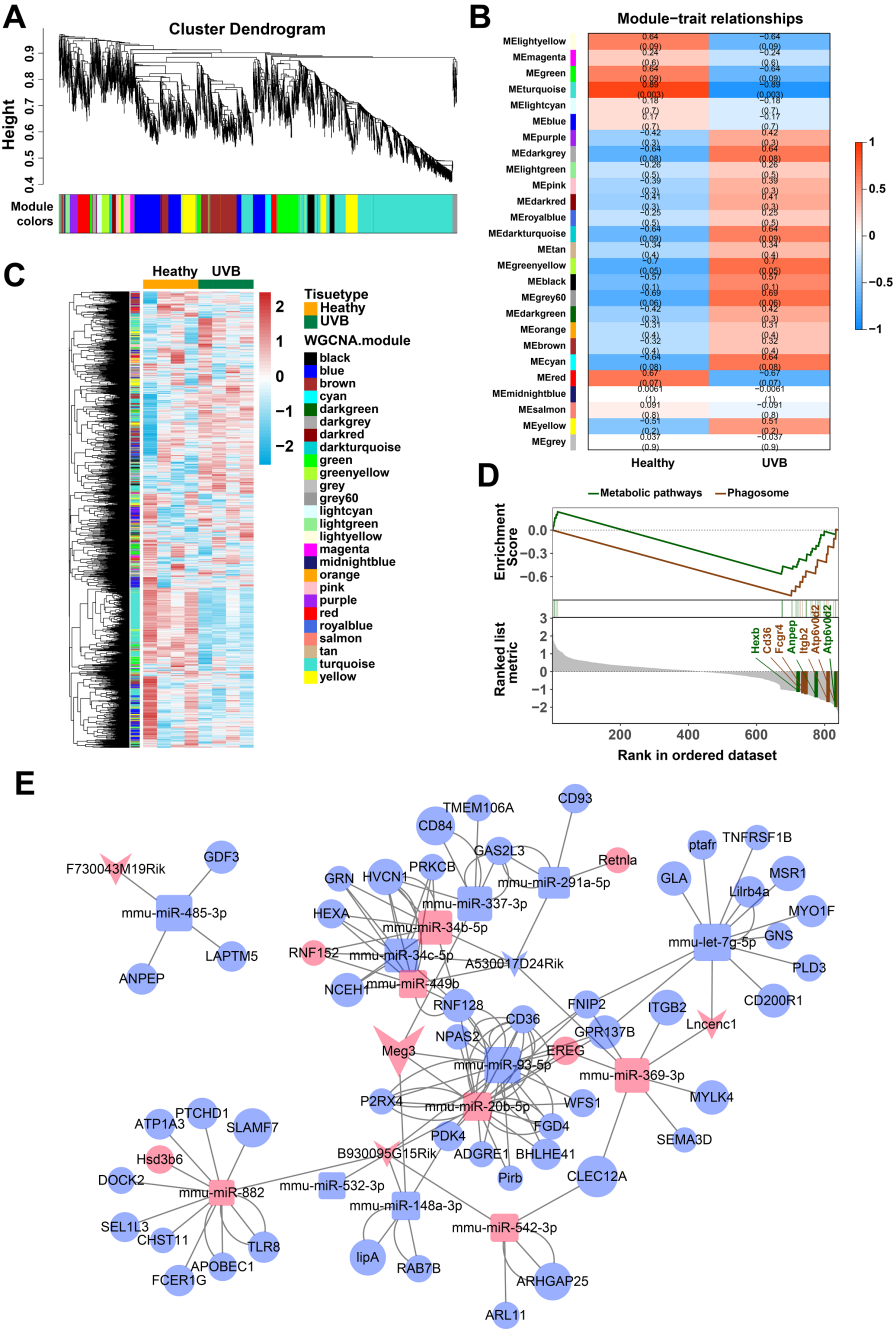
(**A**) Clustering dendrogram of genes by WGCNA. The dissimilarity of genes is based on topological overlap. The genes are assigned to different modules and are identified using different colors. (**B**) The relationship of each WGCNA co-expression modules between normal and UVB irradiated samples were investigated. (**C**) Heatmap of clustered genes in the co-expression modules. The different colors on the horizontal and vertical axis represent different groups and modules. The colors in the middle represents the relativity among each module. (**D**) The significant KEGG-GSEA pathways in differentially expressed mRNAs linked to lncRNAs. The horizontal axis represents the rank in all the ordered dataset, the vertical axes represent enrichment score and ranked list metric. (**E**) The lncRNA-mRNA-miRNA ceRNA network was constructed by five lncRNAs, 14 miRNAs and 54 mRNAs for UVB induced skin lesions.

The weighted gene co-expression network analysis (WGCNA) was used to identify the gene modes which are related to the top 25% of the expressed mRNAs and lncRNAs (Figure 2A) [46]. The threshold for the determination of co-expression gene modes was fixed at a soft power of 9 and a module size cut-off of 30, respectively. The module-trait (healthy and UVB irradiated) co-expression similarity and adjacency analysis were performed in 26 identified gene modules. Turquoise module and green-yellow module demonstrated a significant relationship with healthy and UVB irradiated group, respectively (Figure 2B). The gene expression profiles of each sample were also clustered by the 26 gene modules. As shown in Figure 2C, the gene expression profiles in turquoise and green-yellow modules significantly differed in the two groups. Typically, 1669 mRNAs and 91 lncRNAs in the turquoise and green-yellow modules were further analyzed by KEGG-GSEA (Gene Set Enrichment Analysis) [47]. The phagosome and metabolic pathways were significantly downregulated in the UVB irradiated group (Figure 2D).

### Construction of lncRNA-miRNA-mRNA ceRNA network

The target predictions of lncRNA-miRNA pairs were retrieved from Starbase, and 432 miRNAs were predicted as potential targets of 15 DE-lncRNAs. The common miRNAs between 105 DE-miRNAs and 432 predicted lncRNA-miRNA interactions were selected to obtain lncRNAs-miRNAs network. The interactions between common miRNAs and target mRNAs were retrieved from the miRTarBase [48], miRDB [49], Targetscan, and Starbase database [50, 51]. The target mRNAs were filtered based on the difference in the expression profiles between the healthy and UVB-irradiated groups, as depicted in Figure 2E. After that, we constructed a lncRNA-miRNA-mRNA ceRNA network by using 5 lncRNAs, 14 miRNAs and 54 mRNAs [52–54]. The color and size of each node represented their expression (up-regulated or down-regulated) and fold changes, respectively. The shape of each node represented their type, i.e., circle for mRNAs, square for miRNAs and V-shape for lncRNAs. By the default microarray annotation files, we could identify 655 lncRNAs, and the location of the Meg3/mmu-miR-93-5p/Ereg axis in the hub position of the entire ceRNA network.

### Meg3 is upregulated in UVB irradiated murine skin and related to inflammatory damage

**Figure 3:**
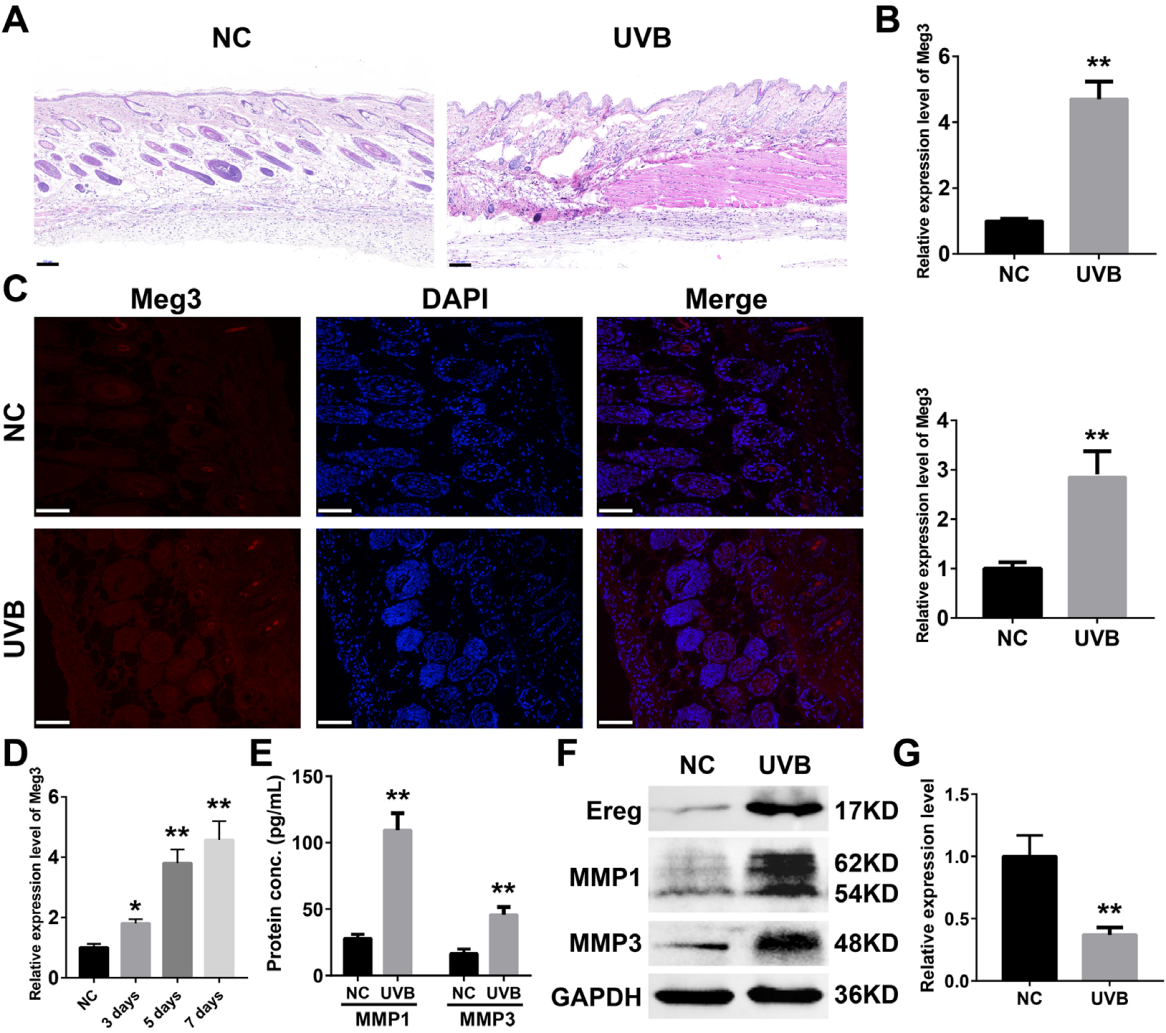
(**A**) H&E staining of murine dorsal skin samples with or without UVB irradiation (scale bar = 100 μm). (**B**) Quantification of Meg3 expression by qRT-PCR in normal and UVB irradiated murine dorsal skin tissues. (**C**) Fluorescence in situ hybridization (FISH) of Meg3 in normal and UVB irradiated murine dorsal skin tissues. (**D**) Quantification of Meg3 expression by qRT-PCR after different exposure time of UVB irradiation. (**E**) Quantification of secreted MMP1 and MMP3 by ELISA in normal and UVB irradiated murine dorsal skin tissues. (**F**) The expression level of Ereg, MMP1, MMP3 by WB in normal and UVB irradiated murine dorsal skin tissues. (**G**) Quantification of miR-93-5p expression by qRT-PCR in normal and UVB irradiated murine dorsal skin tissues.

To further determine expression profiles and potential mechanisms of Meg3 in UVB-irradiated skin tissues, we examined the expression of lncRNA Meg3 in murine skin by using qRT-PCR before and after seven-days of UVB irradiation. Compared to the control tissues, the H&E (hematoxylin and eosin) stained murine skin tissues after seven-days of UVB irradiation exhibited a damaged epidermis, thickened cuticle, and loss of collagen in the dermal layer (Figure 3A). The collagen fibers were disrupted after UVB irradiation, and infiltration of the inflammatory cells were also observed. The qRT-PCR results showed significantly up-regulated expression of the lncRNA Meg3 in UVB-irradiated tissues than those of the control tissues (P < 0.01; Figure 3B) [55]. The fluorescence *in situ* hybridization (FISH) results indicated that the Meg3 expression was activated in the UVB irradiated tissues (P < 0.01; Figure 3C). The detection of Meg3 levels in the murine skin tissues with varying levels of UVB irradiation suggested that the Meg3 expression levels escalated with UVB exposure (Figure 3D). The markers of UVB photo-damage, the expression of MMP1 and MMP3 (matrix metalloproteinase) were also up-regulated in UVB irradiation tissues as detected by ELISA (Enzyme-linked immunosorbent assay, Figure 3E) and western blotting (WB, Figure 3F) analysis. After the UVB irradiation, the protein expression of Ereg improved, and mmu-miR-93-5p levels significantly declined (P < 0.01, Figure 3G).

### Meg3 activated the inflammatory cytokines and regulates miR-93-5p/Ereg expression

**Figure 4:**
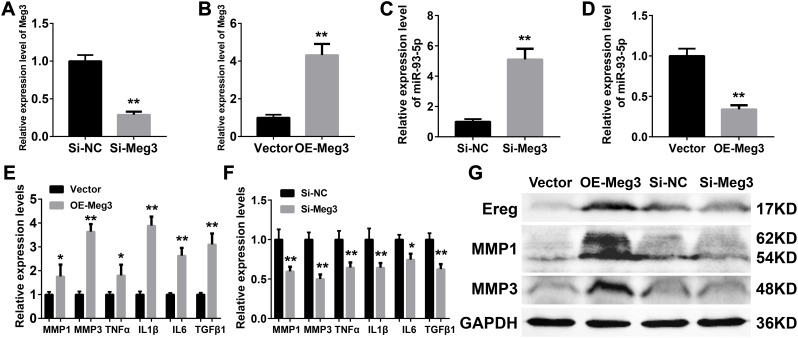
(**A**–**B**) Quantification of Meg3 expression by qRT-PCR after Meg3 siRNA (**A**) or Meg3 overexpression plasmid (**B**) treatment. (**C**–**D**) Quantification of miR-93-5p expression by qRT-PCR after Meg3 siRNA (**C**) or Meg3 overexpression plasmid (**D**) treatment. (**E**–**F**) Quantification of inflammatory cytokines expression by qRT-PCR after Meg3 overexpression plasmid (**E**) or Meg3 siRNA (**F**) treatment. (**G**) WB analysis of Ereg, MMP1 and MMP3 after Meg3 overexpression plasmid or Meg3 siRNA treatment.

Inflammatory responses are an essential pathologic factor of UVB induced skin damage. Skin fibroblast is one of the major participants in the skin inflammation and regeneration after UVB irradiation. The overexpression or RNA interference of Meg3 was performed to determine the function of Meg3 in primary murine skin fibroblasts. The knockdown and overexpression efficiency of Meg3 siRNA and adenoviral vector for Meg3 (OE-Meg3) and siRNA-Meg3, respectively, were confirmed by qRT-PCR (Figures 4A and 4B, respectively). The influence of Meg3 on miR-93-5p expression levels was determined by qRT-PCR. As shown in Figure 4C and 4D, RNA interference of Meg3 resulted in upregulated miR-93-5p, and OE-Meg3 resulted in a declined expression of the miR-93-5p (P < 0.01). The mRNA levels of several inflammatory cytokines were also quantified by qRT-PCR (Figures 4E and 4F), which revealed that the Meg3 overexpression raised the mRNA expression level of MMP1, MMP3, TNF-α, IL1β, IL6, and TGFβ1. RNA silencing of Meg3 suppressed the inflammatory cytokines expressions. Furthermore, the WB analysis showed that Meg3 overexpression elevated the protein levels of Ereg, MMP1, and MMP3; however, Meg3 RNA-silencing did not significantly interfere with the expression of the protein. The protein expression levels in Ereg, MMP1, and MMP3 in null vector and si-NC groups were relatively low; therefore, the changes in protein levels were not observed after the addition of siRNA Meg3. These findings demonstrated that Meg3 not only enhanced the inflammatory response but also stimulated the expression of inflammatory cytokines in the murine skin fibroblasts.

### Meg3 functions as a sponge for miR-93-5p in murine skin fibroblasts

**Figure 5:**
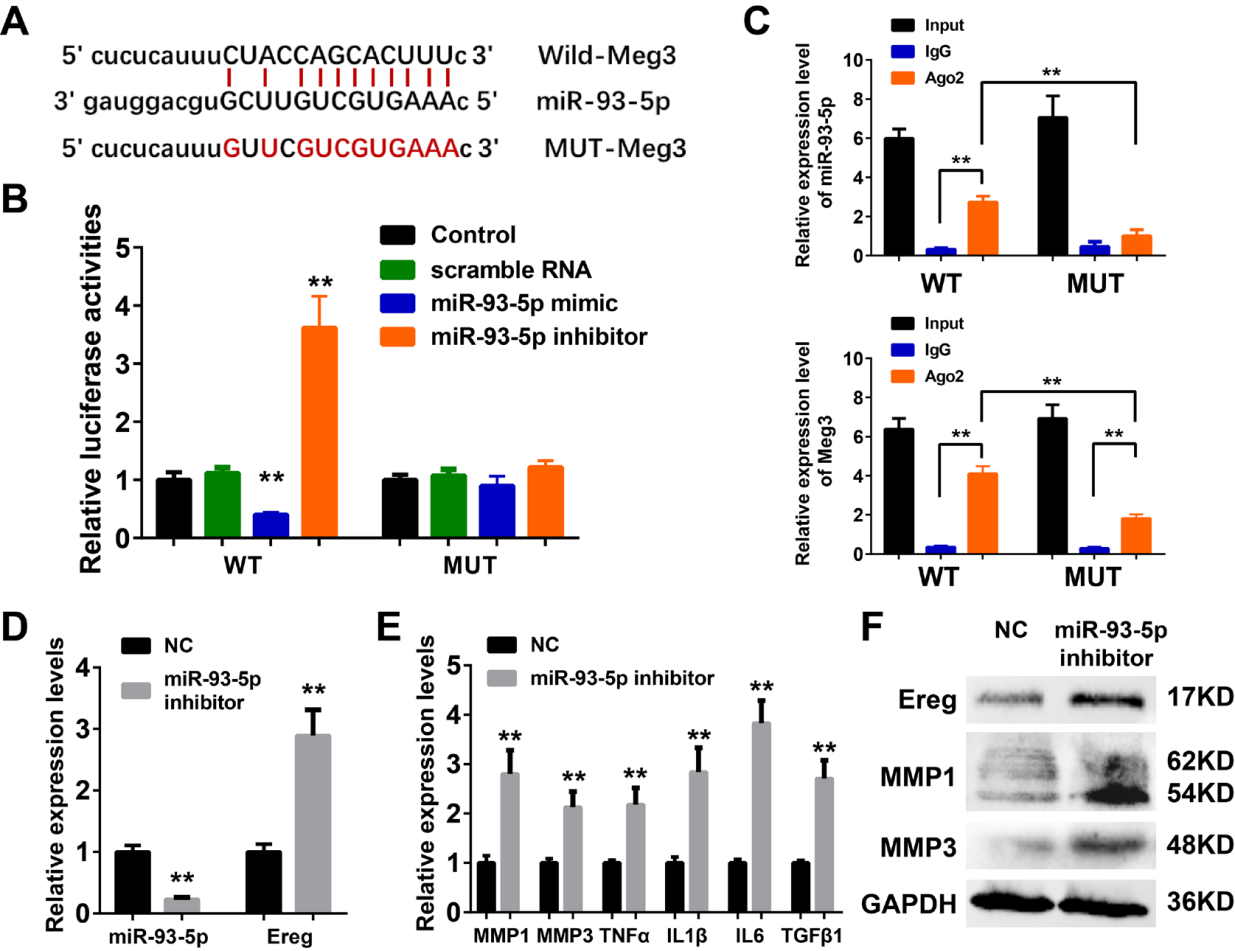
(**A**) The binding region between Meg3 and miR-93-5p were predicted, and the sequences of wild-type Meg3 (WT-Meg3) or mutant Meg3 (MUT-Meg3) sequences were shown. (**B**) The directly binding between Meg3 and miR-93-5p were confirmed by a luciferase reporter assay was performed with the luciferase reporter plasmids of WT-Meg3 or MUT-Meg3. (**C**) RNA immunoprecipitation (RIP) was performed using input from cell lysate, IgG, or anti-Ago2. The relative expression levels of Meg3 and miR-93-5p were detected by qPCR. (**D**) The inhibitory efficiency of miR-93-5p inhibitor and its effects on Ereg expression were determined by qRT-PCR. (**E**) The influences of miR-93-5p inhibitor on the expression of inflammatory cytokines were determined by qRT-PCR. (**F**) The influences of miR-93-5p inhibitor on the protein expression of Ereg, MMP1 and MMP3 were determined by WB.

The ceRNA mechanism played an essential role in the development and regulation of inflammatory skin diseases including solar dermatitis, atopic dermatitis, and psoriasis, etc [56–58]. As reported earlier in the ceRNA network, mmu-miR-93-5p were potential target microRNA of Meg3, and played a vital role in inflammation, cancer, Alzheimer’s disease, and diabetes [59–62]. Therefore, miR-93-5p was chosen as a potential Meg3 candidate for experimental validation. Luciferase reporter assay was used with wild-type and a mutant-type Meg3 reporter, respectively, to determine the direct interaction between lncRNA Meg3 and miR-93-5p (Figure 5A). The luciferase activities of wild-type Meg3 reporter genes were significantly suppressed by miR-93-5p mimic and elevated by miR-93-5p inhibitor, respectively. No significant changes in luciferase activities of the mutant-type Meg3 reporter genes were observed after its’ treatment with miR-93-5p mimic or inhibitor (Figure 5B). As an important part of RNA-induced silencing complexes (RISCs), ncRNAs often performed gene post-transcriptional gene regulatory functions via RISCs. Typically, the microRNAs directly binds with target RNAs that result in RNA degradation *via* an ago2-mediated pathway [23, 24]. As shown in Figure 5C, qRT-PCR analysis of RIP assay (RNA immunoprecipitation) demonstrated that the miR-93-5p expression significantly increased the anti-ago2 group having a wild-type Meg3; and, the difference between IgG group and anti-ago2 group with mutant-type Meg3 were also prominent. As compared to wild-type Meg3, the mutant-type Meg3 levels significantly declined in the anti-ago2 group. These results suggested that miR-93-5p was a direct target miRNA of lncRNA Meg3, and their interactions result in RISC-mediated degradation *via* an ago2-dependent manner.

### MiR-93-5p inhibitor promoted inflammatory response by targeting Ereg in murine skin fibroblasts

As depicted in Figure 5D, a synthetic miR-93-5p inhibitor remarkably suppressed the RNA levels of miR-93-5p and increased the mRNA levels of Ereg, respectively. The bioactivities of miR-93-5p inhibition were validated by qRT-PCR assay and WB analysis of the inflammatory cytokines. The mRNA levels of MMP1, MMP3, TNFα, IL1β, IL6, and TGFβ1 were elevated after miR-93-5p inhibitor incubation (Figure 5E). The protein expression levels of Ereg, MMP1, and MMP3 were also increased in miR-93-5p inhibitor-treated group. In summary, the inhibition of miR-93-5p stimulated the expression of inflammatory cytokines in murine skin fibroblasts.

**Figure 6:**
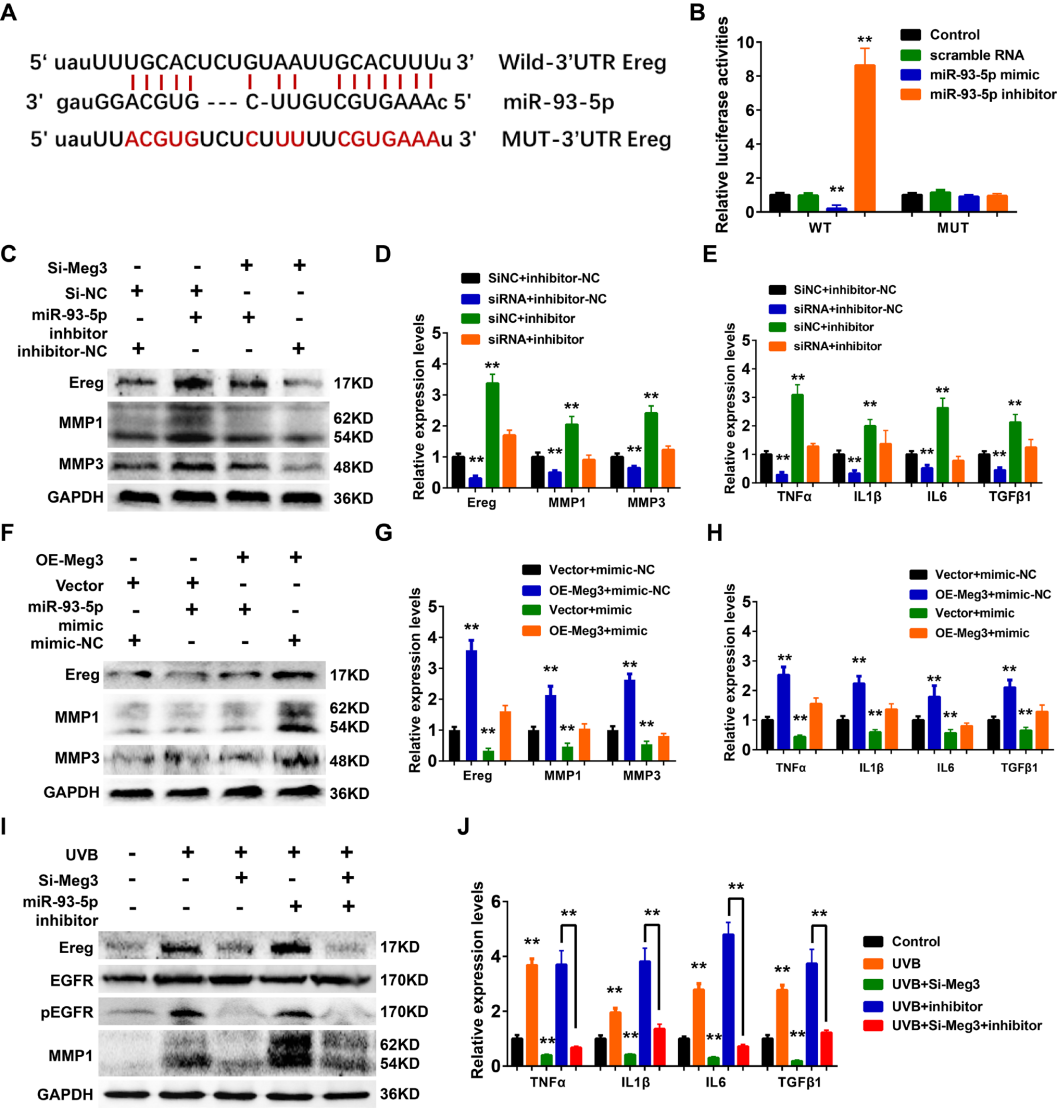
(**A**) The binding region between Ereg and miR-93-5p were predicted, and the sequences of wild-type Ereg (WT-Ereg) or mutant Ereg (MUT-Ereg) sequences were shown. (**B**) The directly binding between Ereg and miR-93-5p were confirmed by a luciferase reporter assay was performed with the luciferase reporter plasmids of WT-Meg3 or MUT-Meg3. (**C**) The protein expression of Ereg, MMP1 and MMP3 were determined by Western blot. Cells were transfected with miR-93-5p inhibitor or siRNA Meg3. (**D**) The inhibitory efficiency of miR-93-5p inhibitor and/or siRNA Meg3 for their effects on Ereg, MMP1 and MMP3 expression were determined by qRT-PCR. (**E**) The inhibitory efficiency of miR-93-5p inhibitor and/or siRNA Meg3 for their effects on inflammatory cytokines expression were determined by qRT-PCR. (**F**) The protein expression of Ereg, MMP1 and MMP3 were determined by Western blot. Cells were transfected with miR-93-5p mimic or pcDNA-Meg3 plasmid. (**G**) The effects of miR-93-5p mimic and/or Meg3 overexpressed plasmid on Ereg, MMP1 and MMP3 expression were determined by qRT-PCR. (**H**) The effects of miR-93-5p mimic and/or Meg3 overexpressed plasmid on inflammatory cytokines expression were determined by qRT-PCR. (**I**) The protein expression of Ereg, EGFR, pEGFR and MMP1 were determined by Western blot. Cells were transfected with miR-93-5p inhibitor and/or siRNA Meg3 after UVB irradiation. (J) The effects of miR-93-5p inhibitor and/or siRNA Meg3 on inflammatory cytokines expression were determined by qRT-PCR.

In the constructed ceRNA network, Ereg was predicted as a potential target of miR-93-5p in Targetscan, Starbase, miRTarBase, and miRDB database. As shown in Figure 6A, the miR-93-5p binding site is located in the 3’UTR of Ereg gene. In the luciferase reporter assay, the mRNA levels of Ereg were determined by the qRT-PCR analysis. In the wild-type Ereg group, Ereg was suppressed by miR-93-5p mimic and elevated by miR-93-5p inhibitor, respectively. No significant changes were observed in the mutant-type Ereg group after miR-93-5p mimic or inhibitor treatment (Figure 6B).

Ereg was first identified from the cultured medium of fibroblast-derived tumor cell lines and directly bound to activate EGFR, and ERBB4 [63–65]. Ereg was reported to interfere the proliferation and regeneration of several organs, including colon, liver, and salivary glands. It could also control inflammation and immune-related signaling pathways which were involved in the pathological processes of renal fibrosis, rheumatoid arthritis, and cancers. In healthy tissues, the expression levels of Ereg were reported to be low; whereas, they were up-regulated in cancer and inflammatory diseases [66–69].

WB results showed that Ereg, MMP1, and MMP3 expression increased after miR-93-5p inhibitor treatment, and the addition of siRNA-Ereg could reverse the up-regulated expression of these proteins (Figure 6C). qRT-PCR results of inflammatory cytokines demonstrated that miR-93-5p inhibitor elevated mRNA levels of Ereg, MMP1, MMP3, TNFα, IL1β, IL6, TGFβ1, and siRNA-Meg3 which played an antagonistic role to a miR-93-5p inhibitor (Figure 6D and 6E). Besides, the overexpression of adenovirus significantly increased the mRNA and protein levels of Ereg, MMP1, and MMP3 (Figure 6F and 6G). Similar results were also prominent in the qRT-PCR results of other inflammatory cytokines, including TNFα, IL1β, IL6, and TGFβ1 (Figure 6H). As expected, the miR-93-5p mimic suppressed the inflammatory effects of Ereg overexpression. In summary, the UVB irradiation stimulated lncRNA Meg3 expression, which activated Ereg expression, and resulted downstream EGFR phosphorylation levels increased, and activation of the inflammatory cytokines *via* acting as the miR-93-5p sponges (Figure 6I). With the addition of siRNA-Meg3 and miR-93-5p inhibitor, these effects were either suppressed or stimulated. The qRT-PCR results of several inflammatory cytokines confirmed the protective potencies of siRNA-Meg3 and inflammatory activities of miR-93-5p inhibitor in UVB-irradiated murine fibroblasts (Figure 6J).

### SiRNA-Meg3 suppresses UVB-induced murine skin lesion in vivo

**Figure 7:**
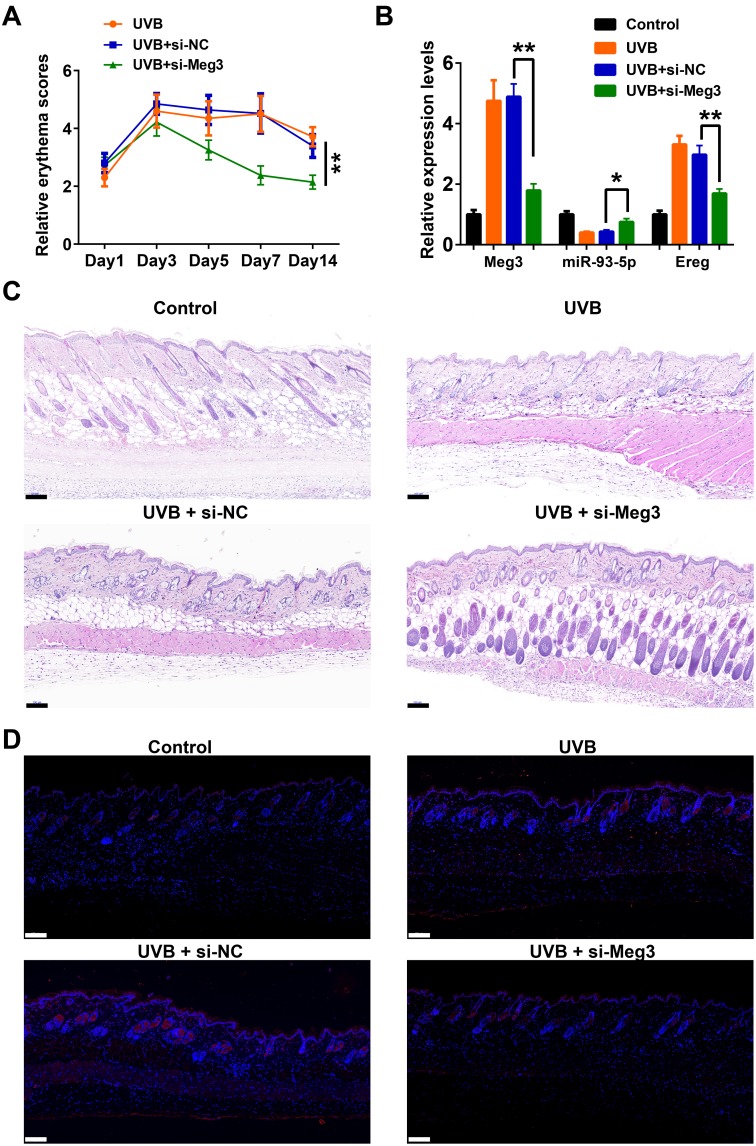
**Meg3 siRNA alleviates UVB-induced skin lesions in a mouse model.** (**A**) Measurement of average erythema score of the murine dorsal skin with or without UVB irradiation. Values presented as mean ± SD. (**B**) Relative expression levels of Meg3, miR-93-5p and Ereg were determined by qRT-PCR. (**C**) H&E staining of the murine dorsal skin sample after UVB irradiation with or without si-Meg3 treatment (scale bar = 100 μm). (**D**) FISH of Meg3 in the murine dorsal skin sample after UVB irradiation with or without si-Meg3 treatment (scale bar = 100 μm).

**Figure 8:**
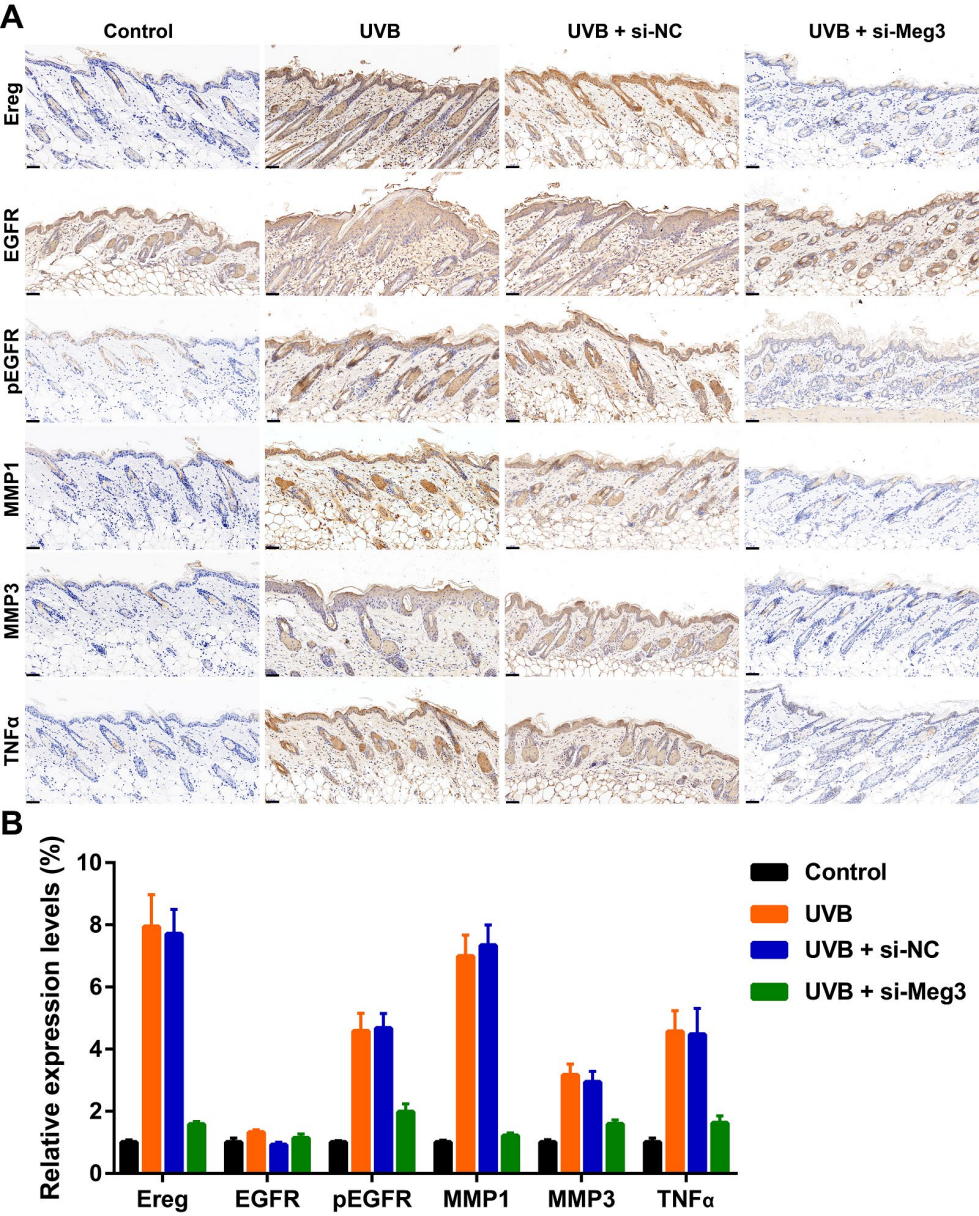
(**A**) Immunohistochemical staining for Ereg, EGFR, pEGFR, MMP1, MMP3 and TNFα in skin samples (scale bar = 50 μm). (**B**) The quantification analysis of relative expression levels.

The protective effects of siRNA-Meg3 in UVB-irradiated skin lesion *via* sponging with miR-93-5p/Ereg prompted us to further validate this ceRNA mechanism in a murine UVB photodamaged model of murine skin. The siRNA-Meg3 was loaded in a cationic liposome and intravenously injected to nude mice, followed by 14-days of continuous UVB irradiation. Erythema scores on the dorsal skin of mice reached peaks within 3–7 days of UVB and UVB exposure plus si-NC groups. In the si-Meg3 treated group, there were lower erythema scores than that of UVB or UVB plus si-NC groups (Figure 7A). qRT-PCR results of Meg3, Ereg, and miR-93-5p showed that the UVB irradiation significantly inhibited miR-93-5p expression and increased the expression of Meg3 and Ereg, respectively. Moreover, the addition of si-Meg3 partially reversed the declined miR-93-5p and elevated Ereg expression after the UVB irradiation (Figure 7B). The H&E (hematoxylin and eosin) staining of the murine dorsal skin tissue confirmed that the UVB irradiation led to inflammatory changes, including increased epidermis thickness, hyperkeratosis, and hyperplastic sebaceous glands. The administration of siRNA-Meg3 partially reversed these morphological changes, which further confirmed the protective potential of the UVB-induced photodamage (Figure 7C). FISH analysis of lncRNA Meg3 demonstrated significant differences in the Meg3 levels between the control, and UVB irradiated groups, as well as the si-NC, and siRNA-Meg3 treated groups, respectively (Figure 7D). The overall distributions of Meg3 did not overlap with DAPI, which suggested Meg3 was probably located in the cytoplasm or extracellular matrix. We further determined the expression levels of Ereg, EGFR, pEGFR, MMP1, MMP3, and TNFα in each group by immunohistochemistry (Figure 8). As compared with the *in vitro* experiments, the expression of Ereg, pEGFR, MMP1, MMP3, and TNFα were significantly elevated in the UVB-irradiated group. No other changes were noticed in EGFR expression in all the groups; the decreased expression levels of Ereg, pEGFR, MMP1, MMP3, and TNFα in the si-Meg3 group varied from those of the si-NC group. These results were consistent with those of the UVB-irradiated murine fibroblasts, which revealed that the Meg3 mediated inflammatory response *via* sponging miR-93-5p/Ereg axis after the UVB irradiation.

## DISCUSSION

With the destruction of the ozone layer, the risk of skin damage due to increasing levels of ultraviolet radiation is alarming [ref]. Ultraviolet radiation can cause skin diseases by inducing DNA damage to the skin cells, oxidative stress, and programmed cell death [70]. According to the literature, lncRNA plays a vital role in the inflammatory responses, i.e., it controls the expression level changes of the immune cells during their differentiation, which affect other immune responses [71–74]. LncRNAs also control the inflammatory response by directly acting on the expression of inflammatory factors. LncRNAs participate in the immune and inflammatory pathways *via* a variety of mechanisms which plays a vital role in many skin diseases such as psoriasis, eczema, and photodamage. Sonkoly et al. reported that lncRNA-PRINS was overexpressed in the lesion tissues of psoriasis patients [75]. LncRNA-PRINS could increase the sensitivity of keratinocytes to spontaneous apoptosis by up-regulating the expression of GIP3, which promoted the proliferation of keratinocytes, and affected the development of psoriasis. Qiao et al. reported that lncRNA MSX2P1 could suppress miR-6731-5p in IL-22 stimulated keratinocytes which activated the expression of S100A7, IL-23, NFκB and TNFα [76]. LncRNA Meg3 located at chromosome 14q32 could encode a 1.5kb length transcript related to various human cancers. For the non-cancer diseases, Braconi et al. reported that overexpressed Meg3 significantly suppressed cell proliferation and induced apoptosis in human liver cancer cells. He et al. reported that Meg3 stimulated the pathogenesis and progress of liver fibrosis by activating HSC *via* a TGF-β1 dependent manner [77]. Furthermore, the expression level of Meg3 declined in fibrotic liver tissues after carbon tetrachloride (CCl_4_) treatment. Recently, Zhao et al. reported that Meg3 activated apoptosis in myocardial cells in hypoxic atmosphere mediated by FoxO1 pathway [78] Zha et al. reported that Meg3 sponged miR-181a/Egr-1/TLR4 signaling axis and activated inflammatory responses in diabetic nephropathy [33]. To our knowledge, competed endogenous RNA was one of the most important mechanism of lncRNA to regulate target gene expression in inflammatory response and cancer. For lncRNA Meg3, there was a panel of reports of its biological functions *via* the ceRNA mechanism. Recently, Zhang et al. reported that Meg3 suppressed laryngeal cancer cell proliferation *via* sponging miR-23a/APAF-1 axis [79]. Chen et al. reported that Meg3 alleviated ECM degradation in osteoarthritis chondrocytes by targeting miR-93/TGFBR2 axis [80]. Huang et al. reported that Meg3 could directly bind with miR-27a as a ceRNA and activate PHLPP2 expression to inhibit bladder cancer cells invasion [34]. Meg3 also affected the Treg/Th17 balance in asthma patients *via* miR-17/RORγt axis [81]. Although there was a panel of reports related to Meg3-mediated inflammatory responses, the role and mechanism of Meg3 in inflammatory skin disease and UVB-related damage remained unclear.

In the current study, lncRNA Meg3 was predicted as an inflammation mediator in the lncRNA reannotation of microarray data in UVB irradiated murine dorsal skin tissues. The WGCNA and ceRNA network analysis predicted that Meg3 sponged miR-93-5p and epiregulin by a lncRNA-miRNA-mRNA competed endogenous mechanism. Both qRT-PCR and FISH results validated that Meg3 was upregulated in UVB irradiated murine skin tissues. As one of the main component in the extracellular matrix of skin tissue, collagen was usually degraded by MMP1/3 under inflammatory or cancer microenvironment. Our results also suggested that MMP1 and MMP3 were useful markers for UVB-induced skin tissue injury. MMP1, MMP3, and Ereg were overexpressed after UVB irradiation at RNA and protein levels, respectively. The overexpressed proteins could be reversed by siRNA-Meg3 in primary murine skin fibroblasts. Both luciferase and RNA-pulldown assays indicated that the direct binding of miR-93-5p with Meg3 and Ereg, respectively. Our results further suggested that the UVB induced inflammatory skin lesions were dependent on Meg3/miR-93-5p/Ereg axis by a ceRNA mechanism.

In conclusion, our results suggested that lncRNA reannotation, bioinformatics analysis, and ceRNA network construction were valid predicted methods for discovering novel lncRNAs related to the identified biological functions and mechanisms. The current research revealed that lncRNA Meg3 upregulated Ereg mRNA levels by sponging miR-93-5p function *via* ceRNA mechanism. LncRNA Meg3 stimulated inflammatory responses after UVB irradiation both *in vitro* and *in vivo*. The RNA silence of Meg3 relieved UVB-induced photodamage by suppressing the MMP1/3 expression and inflammatory cytokine activation. Our results suggested that lncRNA Meg3 could potentially act as a biomarker and therapeutic target for UVB-induced skin injury. These results also provide a basis for the combination of bioinformatics, data mining, and experimental validation as an effective protocol to discover bioactive lncRNAs in diverse diseases.

## MATERIALS AND METHODS

### LncRNA reannotation

LncRNA annotations were collected from two sources, i.e., the catalog of lncRNAs from the Gencode database (Mus musculus GRCm38.p6) and the catalog of lncRNAs generated based on the transcriptome assembly from RNA-seq data. Once such transcript of LncRNA overlapped on the same strand between these two sources, and therefore, the Ensembl annotation was kept to avoid redundancy. The probes that were mapped uniquely to the genome with no mismatch were kept; other probes that were mapped to protein-coding transcripts or pseudogene transcripts were removed. We obtained probes, and corresponding lncRNA genes with at least three probes by matching the rest of the probes to the ncRNA sequences. The lncRNA expression was quantile normalized across different biological samples.

### Cell culture and UVB radiation

The primary murine skin fibroblasts were obtained according to the previous reports [82–84] and cultured in Dulbecco’s Modified Eagle’s Medium supplemented with 10% fetal bovine serum and 1% penicillin-streptomycin (Life Technologies) in 5% CO_2_ at 37 °C. The cells were washed and covered with a thin layer of PBS for UV irradiation. Mock-irradiated controls without UVB irradiation followed the same schedule of medium changes. For irradiation, a UVB lamp was used to deliver uniform irradiation at a distance of 15 cm, and the radiation intensity was monitored using a UVB light meter. The irradiation dosage of 100 mJ/cm^2^ was used, the cells were washed twice by PBS buffer and then incubated with DMEM culture after UVB irradiation.

### ELISA and Western blot assays

An ELISA kit (Raybiotech, Inc.) was used to detect the secretion of MMP-1 and MMP-3 following the manufacturer's protocol. Proteins were extracted using a radioimmunoprecipitation assay buffer (Beyotime Institute of Biotechnology). Proteins quantified by a BCA protein assay kit were separated by 8%–10% SDS–PAGE, and then transferred onto polyvinylidene fluoride membranes (Millipore). The membranes were blocked with nonfat milk at room temperature for 2 h. The membranes were then incubated with primary antibodies overnight at 4 °C and HRP-conjugated secondary antibodies (Beyotime Institute of Biotechnology) for 1 h at room temperature. An enhanced chemiluminescence reagent (Beyotime Institute of Biotechnology) was used to visualize the blots. The band densities were analyzed using the software ImageQuant (Bio-Rad, ChemiDoc MP).

### Animal study

All animal experiments were performed following the protocol approved by the Institutional Animal Care and Use Committee of the West China Hospital. Female Balb/c nude mice (6 weeks) were purchased from the Animal Center of Sichuan University (China). Mouse dorsal skin (each group, n = 6) was exposed to UVB irradiation at 300 mJ/cm^2^ according to our previous reports.^55^ Samples were given intravenous injection using a vehicle or siRNA-Meg3 lipoplex followed by UVB irradiation thrice a week for two weeks. Erythema in the skin of mice was evaluated following established protocols, and the mean grades were calculated at each time point after UVB irradiation. The brief protocol of lipoplex preparation was described below, DOTAP (dioleoyl trimethylammonium propane) and cholesterol (molar ratio 1:1) were dissolved in chloroform, and then the solvent was evaporated to form a lipid film. The lipid film was rehydrated in 5% Dextrose at 60 °C for 30 min, and the dispersed by a bath sonicate at 60 °C for 10 min to form cationic liposomes. The scramble siRNA or si-Meg3 were incubated to the cationic liposomes (w/w 1:20 or 1:30) at 37 °C for 10 min. Finally, the lipoplex was extruded through a 100 nm polycarbonate filter by using a Mini-Extruder instrument.

### Ethical approval

All procedures involving animals were performed in compliance with guidelines of the Chengdu University of Traditional Chinese Medicine.

## Supplementary Material

Supplementary Materials

Supplementary Figure 1
