# Supplementary material for: Competing endogenous network analysis identifies lncRNA Meg3 activates inflammatory damage in UVB induced murine skin lesion by sponging miR-93-5p/epiregulin axis
Source: Aging (Albany NY). 2019 Nov 24;11(22):10664–83. doi: 10.18632/aging.102483 (PMC6914409; doi:10.18632/aging.102483)
Supplement: Supplementary Materials [file aging-11-102483-s001..pdf]

SUPPLEMENTARY METHODS

Microarray data processing and visualization

The microarray dataset GSE80427 and GSE80428 was downloaded from the Gene Expression Omnibus which contains eight paired UVB-injured and adjacent murine skin tissues samples. Profile data extractions were performed based on the R and Bioconductor environment. GSEA was performed using GSEA 2.2.1 (http://www.broadinstitute.org/gsea).

Histological analysis

Samples were fixed in 4% paraformaldehyde in PBS for 24 h, washed with tap water, dehydrated with graded ethanols, and embedded in paraffin wax. Blocks of paraffin wax were cut in 4 μm sections, mounted on glass slides, dewaxed, rehydrated through graded ethanols, and H&E-stained, incubated by corresponding primary antibody or FISH probe. Analyses were performed using a light microscope (Olympus, Japan). Skin samples were fixed with 10% neutralized formalin, embedded in paraffin, and used for immunohistochemistry or immunofluorescence staining using a rabbit polyclonal antibody as described previously.

Statistical study

Experiments were run independently in triplicate. Data are presented as means ± standard deviation (or standard error of values) obtained from the experiments. The student's t-test was usually used to determine if two sets of data (follow normal distributions) were significantly different from each other. If the data did not follow a normal distribution, we should perform a non-parameter method, such as a Mann-Whitney U test, instead of a student's t-test. If there were more than two sets of data which follow normal distributions, one-way ANOVA (one-way analysis of variance) was commonly used to determine whether the data sets were different from each other.
