# Supplementary material for: Competing endogenous network analysis identifies lncRNA Meg3 activates inflammatory damage in UVB induced murine skin lesion by sponging miR-93-5p/epiregulin axis
Source: Aging (Albany NY). 2019 Nov 24;11(22):10664–83. doi: 10.18632/aging.102483 (PMC6914409; doi:10.18632/aging.102483)
Supplement: Supplementary Figure 1 [file aging-11-102483-s002..pdf]

SUPPLEMENTARY FIGURE

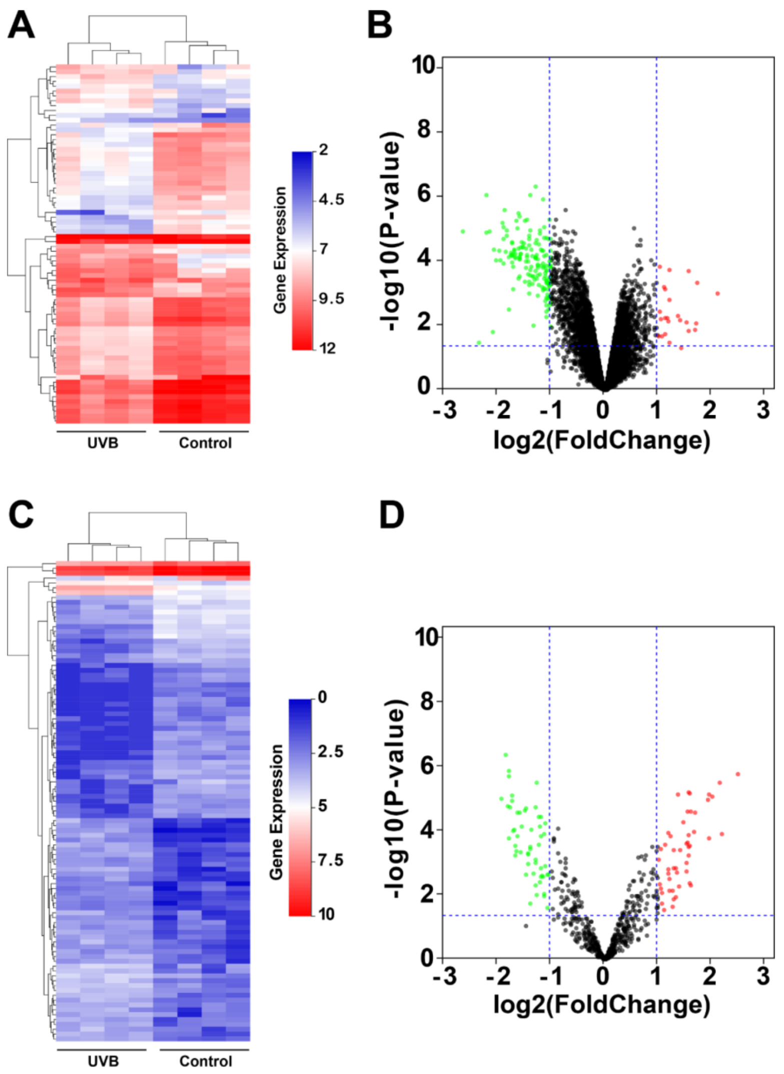

Supplementary Figure 1. Bioinformatics analysis of mRNA and miRNA of murine skin samples after UVB irradiation. (**A**) the cluster of all genes by Hierarchical clustering method; (**B**) Volcano plot of all detected genes by using the Log_2FC and $-Log_{10}$ P-values as cut-off; (**C**) the cluster of all miRNAs by Hierarchical clustering method; (**D**) Volcano plot of all detected miRNAs by using the Log_2FC and $-Log_{10}$ P-values as cut-off.
